# Evaluation of the Appropriate Age Range of Colorectal Cancer Screening Based on the Changing Epidemiology in the Past 20 Years in Taiwan

**DOI:** 10.5402/2012/960867

**Published:** 2012-08-30

**Authors:** Huan-Cheng Chang, Jorng-Tzong Horng, Wen-Chu Lin, Hsin-Wen Lai, Cheng-Wei Chang, Tzu-An Chen

**Affiliations:** ^1^Department of Family Medicine, Taiwan Landseed Hospital, Ping-Jen City, Taoyuan 32449, Taiwan; ^2^Department of Health Care Management, Chang Gung University, Taoyuan 33302, Taiwan; ^3^Department of Computer Science and Information Engineering, National Central University, Taoyuan 32001, Taiwan; ^4^Division of Gastroenterology, Department of Internal Medicine, Shin Kong Wu Ho-Su Memorial Hospital, Taipei 11101, Taiwan; ^5^Department of Information Management, Hsing Wu College, New Taipei City 24452, Taiwan; ^6^Department of Surgery, Taiwan Landseed Hospital, Ping-Jen City, Taoyuan 32449, Taiwan

## Abstract

*Introduction*. According to the recommendation of the United States Preventative Services Task Force, most countries provide average-risk screening for colorectal cancers (CRCs) between the ages of 50 and 75 years. However, the age range of screening should be modified because of an increasing life span. *Methods*. Totally 124,314 CRC cases were registered in Taiwan Cancer Registry from 1988 to 2007. The 20-year study period was divided into four 5-year increments. We divided the patients into four age groups (under age 50, age 50–74, age 74–84, and over age 85) in each increment to determine whether there were changes in the age distribution. *Results*. In the subgroup of patients under age 50, the number of CRC cases increased, but they accounted for a decreasing proportion of the total CRCs. In the 50–74 age group, the proportion of CRC cases also dropped. In contrast, the proportion increased in the 75–84 age group. Therefore, 43.63% of CRC patients would not be delegated to screen in the period of 2003–2007 if the CRC screening were restricted in the 50–74 age group. *Conclusions*. CRC screening for healthy individuals aged over 75 years is necessary.

## 1. Introduction 

Colorectal cancer (CRC) is a worldwide health problem, particularly in developed countries. According to the report by the Bureau of Health Promotion, CRC is the most common malignancy in Taiwan, and the age-standardized incidence rate was 37.1 per 100,000 people in 2007 [1]. Although the age-standardized incidence rates of CRC are lower among women than among men, the lifetime probability of developing CRC is similar because women live longer. In the United States, the lifetime risk of CRC is 5.12% for both men and women born today [2]. 

Since 1995, the United States Preventative Services Task Force (USPSTF) has recommended that screening begin at age 50 for average-risk persons [3]. The USPSTF recommends screening for colorectal cancer (CRC) using fecal occult blood testing, sigmoidoscopy, or colonoscopy, in adults, beginning at age 50 years and continuing until age 75 years. On the other hand, the USPSTF recommends against routine screening for colorectal cancer in adults age 76 to 85 years [4, 5]. The purpose of this study was to determine the appropriate age for screening for CRC in Taiwan. Recent studies have reported an increasing number of CRC resections in patients younger than 50 [6]. Among all CRC cases, the proportion of older patients has also increased [7]. In addition, the age at which women develop CRC is increasing [8]. To maximize the effects of screening, it is necessary to expand the age range and to take into account gender differences. We analyzed the age of CRC patients in Taiwan for the past 20 years and assessed the appropriateness of the current recommended screening age and methods. 

## 2. Methods

This study was based on the Taiwan Cancer Registry, which was created by the Bureau of Health Promotion, Department of Health, Taiwan. This anonymized database is released for research, and the personal information such as names or addresses was excluded. Our study was approved by Landseed Institutional Review Board. The inform consents were not obtained because the data were analyzed anonymously. For cancer prevention and control work in Taiwan, the Department of Health established a cancer registration system in 1979. Clinicians are requested to report the diagnosis and treatment information for new cancer cases. A total of 124,314 CRC cases registered from 1988 to 2007 were enrolled in our study. The International Classification of Diseases, Clinical Modification (ICD-9-CM, ICD for short), was used to assign codes to diagnoses of CRC. All cases were characterized by sex, age at diagnosis, and ICD number. The population composition data were obtained from the Statistical Yearbook of the Interior (http://sowf.moi.gov.tw/stat/year/list.htm), officially published by the Ministry of the Interior, Taiwan.

The 20-year study period was divided into four 5-year increments. The screening age range suggested by the USPSTF is 50 to 75-years-old, and persons over 85 years old are not advised to undergo screening. We divided the patients into four age groups (under age 50, age 50–74, age 75–84, and over age 85) to evaluate whether there were changes in the age distribution of CRC over time.

## 3. Results 

**Figure 1:**
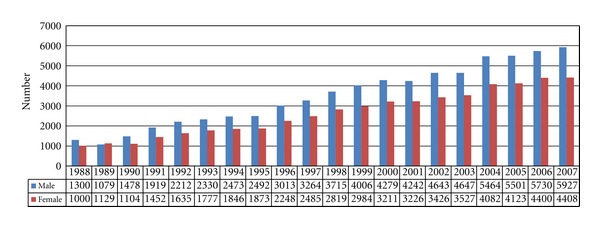
Number of CRC cases from 1988 to 2007.

**Figure 2:**
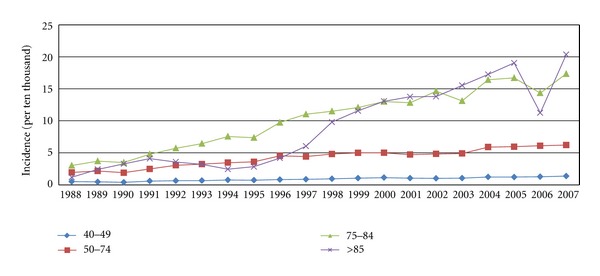
The age-specific incidence of CRC in Taiwan.

In the 20-year study period, the number of the CRC cases increased each year (Figure 1). The CRC incidence for each age group over the past 20 years was showed in the Figure 2. Because the incidence of CRC under age 40 was extremely rare, we only calculated the incidence of age 40–49 but not the incidence of under age 50. The incidence rates in the groups of age 40–49 and age 50–74 remained more stable than those in the groups of age 75–84, and over age 85.

In the subgroup of patients younger than 50-years-old, the CRC case numbers increased, but these cases accounted for a decreasing proportion of total CRCs (Table 1). The proportion also dropped in the 50–74 age group. In contrast, the proportion increased in the 75–84 age group. The proportion also increased in the group over age 85, although the number of cases was small.

## 4. Discussion 

The proportion of CRCs in the 50–74 age group, that is, the screening age suggested by the USPSTF, among all CRCs decreased in the past 20 years. In the same period, the proportion increased for the 75–84 age group. 

Taiwan's National Health Insurance (NHI) is a universal health insurance program that covers all comprehensive services. 97 percent of the total eligible population had enrolled in the NHI. The 3 percent not enrolled may be living overseas or in very remote areas and perhaps includes the near-poor with irregular income sources or independent-minded, wealthy self-employed people. We believed nearly total CRC cases in Taiwan were included in the registry we used for this study because it involved the payment of medical expenses. 

From 1988 to 2007, the population of Taiwan increased from 19,954,397 to 22,958,360. The proportion of older individuals also increased. In 1988, the population aged over 65 years was 1,145,787. In 2007, the population doubled to 2,343,092. Because the incidence of CRC increases with age and the elderly population has increased, the number of cases markedly increased. The cause of the yearly increase in CRC cases could be mostly derived from longer lifespans. We observed a relatively steady incidence in the 40–49 and 50–75 age groups (Figure 2). This finding could be regarded as indirect evidence of the accuracy of our data. If the registry have not been complete in the early years of our study period, the age-specific incidence rate should have increased in all age groups in the later years. Because the increasing incidence rates were only noted in the groups of older individuals but not for all age groups; our result reflects a real increase in the number of CRCs in Taiwan and not just better registration. The CRC screening project in Taiwan started in 2004 and involves checking for fecal occult blood in people aged 50–69 years every two years. Few people were enrolled. The medical authorities diligently tried to expand the screening project, but fewer than 300,000 persons were screened in 2008. Our data may be regarded as a true epidemiological result not affected by the screening program because most people did not participate in the screening program in Taiwan. 

Screening for CRC reduces mortality by allowing physicians to detect cancer at earlier stages and to identify and remove precancerous adenomatous polyps (asymptomatic benign precursor lesions that may lead to colorectal cancer). Recent declines in both incidence and mortality could be attributed to large-scale screening [9]. The appropriate age for CRC screening suggested by the USPSTF is 50 to 74 years old. Screening is not routinely recommended in persons older than 75 years of age, and it is not recommended at all in persons older than 85 years of age, even though the risk of CRC and advanced polyps continues to increase with age. If persons between the ages of 75 and 85 have never undergone screening, decisions about screening should be individualized according to health status. This suggestion was based on the MISCAN and SimCRC models, and standardized model profiles are available at http://cisnet.cancer.gov/profiles/. Both models simulate the life histories of a large population of individuals from birth to death. As each individual ages, there is a chance that an adenoma will develop. One or more adenomas can occur in an individual, and each adenoma can independently develop into preclinical (i.e., undiagnosed) CRC. The risk for developing an adenoma depends on age, sex, and baseline individual risk. The models track the location and size of each adenoma; these characteristics influence disease progression and the chance that the adenoma is found by screening. The natural history component of each model was calibrated to 1975–1979 clinical incidence data and adenoma prevalence from autopsy studies in the same period.

Based on our statistics, at present, we might miss almost half of the CRC cases if we only screen the population between ages 50 and 74. In 1988–1992, we could identify approximately two-thirds of CRC cases by screening this age group. In 2002–2007, the proportion of CRC cases for patients between ages 50 to 74 dropped to 58% in males and 56% in females. On the other hand, the proportion of CRCs in individuals age 75 to 84 has increased and now accounts for almost one quarter of the total CRC cases. In 2008, the average life expectancy at birth in Taiwan was 78.97. The male life expectancy at birth was 75.88, and the female life expectancy at birth was 82.46. Compared with the previous year, the male and female life expectancies at birth increased by 0.29 years and 0.52 years, respectively (http://sowf.moi.gov.tw/). Because of the increase in the average lifespan, the incidence of CRC in elderly individuals is very different than it was 30 years ago. In addition, the modern advancement of surgical techniques and chemotherapy agents has also improved the prognosis of CRC [10, 11]. Our question is as follows: can a model established 30 years ago be applied to the present disease course of CRC?

Other scholars have raised the same question. Rozen et al. examined the USPSTF guidelines in Jewish populations using Israel Cancer Registry data for 1980–2008 [12]. In patients aged between 50 and 74 and in those older than 75, there was an increasing incidence of CRC. However, the percentage for CRC patients aged between 50 and 74 years decreased, while it increased for those who were more than 75-years-old. Therefore, 45.3% of patients (39.1% aged ≥75 years) would not have been electively screened under the current guidelines. This finding was compatible with our study. These results should influence CRC screening age guidelines, especially for healthy individuals aged 75 years or more. These results also highlight the need for noninvasive but sensitive screening methods that can be administered before performing a colonoscopy exam. Shellnut et al. designed another study to evaluate the appropriateness of the USPSTF screening recommendations for CRC [7]. A total of 6,925 CRC cases were identified by the Beaumont hospital system tumor registry from 1973 to 2007. Because nearly 50% of the patients surgically treated for CRC fall outside the current screening criteria, they also questioned the recommended age to stop screening that is listed in the current USPSTF guidelines. 

Today, there is a range of options available for CRC screening in the average-risk population. These screening tools can be divided into two categories: stool tests, which include tests for occult blood or exfoliated DNA and structural exams, which include flexible sigmoidoscopy, colonoscopy, double-contrast barium enema, and computed tomographic colonography. The annual FOBT is the only screening method with randomized, controlled trials demonstrating a decrease in colorectal cancer-specific mortality [13]. However, the FOBT is limited in its ability to identify significant premalignant lesions. Colonoscopy has the highest specificity and sensitivity and can be used not only for screening but also for the diagnosis and treatment of lesions throughout the colon. However, the effectiveness of CRC prevention by colonoscopy is still controversial [14]. In 2009, the American College of Gastroenterology published guidelines recommending a colonoscopy every 10 years. For average-risk individuals, the suggested starting age for CRC screening is still 50-years-old because of the potential effectiveness of colonoscopy to reduce the incidence and mortality of CRC [15]. According to our study, the percentage of CRCs in the population under age 50 is decreasing. In addition, the incidence of CRC after age 50 has increased. To maximize the preventative effect, age 50 might be a reasonable starting age for colonoscopy screening in both sexes. 

Our study has some limitations. First, cases of hereditary nonpolyposis colorectal cancer (HNPCC) syndrome and familial adenomatous polyposis (FAP) were not identified in our database. People with HNPCC and FAP have a high risk of colorectal cancers occurring at a young age. These epidemiological characteristics are much different from those of non-HNPCC and non-FAP cases. Second, the survival benefit of screening programs was not studied. For elderly patients with colorectal cancers, the potential risk of current treatment was higher than that for younger patients. Cost is another important factor, but we cannot make further analysis based on our limited materials. The cost of screening and treatment after diagnosing CRC in the elder population should be evaluated in the future studies. 

## 5. Conclusions

In our population-based study, we found that the proportion of CRC in elderly individuals increased over the past 20 years in Taiwan. Because of the longer average lifespan, screening for healthy individuals who are more than 75-years-old is necessary. 

## Figures and Tables

**Table 1:** Proportion of CRC for different age groups in the four study periods.

Age range			1988–1992	1993–1997	1998–2002	2003–2007
<50	Male	*N*	1457	2034	2919	3346
		%	16.97	14.95	13.98	12.27
	Female	*N*	1324	1881	2815	3179
		%	20.95	18.39	17.97	15.48

50–74	Male	*N*	5971	9108	12894	15812
		%	69.53	66.97	61.74	57.99
	Female	*N*	4090	6320	8840	11577
		%	64.72	61.79	56.43	56.37

75–84	Male	*N*	1057	2300	4336	6769
		%	12.31	16.91	20.76	24.82
	Female	*N*	807	1813	3247	4449
		%	12.77	17.72	20.73	21.66

>85	Male	*N*	103	160	736	1342
		%	1.20	1.18	3.52	4.92
	Female	*N*	99	215	764	1332
		%	1.57	2.10	4.88	6.49

Sum	Male	*N*	8588	13602	20885	27269
	Female	*N*	6320	10229	15666	20537
